# Evaluation of Bioactive Compounds, Antioxidant Capacity, and Anti-Inflammatory Effects of Lipophilic and Hydrophilic Extracts of the Pericarp of *Passiflora tripartita* var. *mollissima* at Two Stages of Ripening

**DOI:** 10.3390/molecules29204964

**Published:** 2024-10-21

**Authors:** Hugo Jesús Justil-Guerrero, Jorge Luis Arroyo-Acevedo, Juan Pedro Rojas-Armas, Carlos Orlando García-Bustamante, Miriam Palomino-Pacheco, Robert Dante Almonacid-Román, James Willan Calva Torres

**Affiliations:** 1 Laboratory of Pharmacology, Faculty of Medicine, Universidad Nacional Mayor de San Marcos, Av. Miguel Grau 755, Lima 15001, Peru; jarroyoa@unmsm.edu.pe (J.L.A.-A.); jrojasa1@unmsm.edu.pe (J.P.R.-A.); cgarciab@unmsm.edu.pe (C.O.G.-B.); 2 Laboratory of Biochemistry, Faculty of Medicine, Universidad Nacional Mayor de San Marcos, Lima 15001, Peru; mpalominop1@unmsm.edu.pe; 3 Laboratory of Microbiology, Faculty of Pharmacy and Biochemistry, Universidad Nacional Mayor de San Marcos, Jr. Huanta 1182, Lima 15001, Peru; robert.almonacid@unmsm.edu.pe; 4 Departamento de Química, Universidad Técnica Particular de Loja, San Cayetano Alto s/n, Loja 1101608, Ecuador; jwcalva@utpl.edu.ec

**Keywords:** *Passiflora tripartita* var. *mollissima*, antioxidant, oxidative stress, anti-inflammatory, inflammatory cytokines

## Abstract

Chronic disease inflammation requires safe complementary treatments. The pericarp of *Passiflora tripartita* var. *mollissima* (PTM) contains potential anti-inflammatory metabolites. This study aimed to evaluate the bioactive components, antioxidant capacity, and anti-inflammatory effects of PTM extracts at two ripening stages. The bioactive compounds in the hydrophilic and lipophilic extracts of mature and green pericarps were identified by GC-MS and UV–VIS, while the antioxidant capacity was measured by free radical reduction. Anti-inflammatory effects were tested using a rat paw edema model with carrageenan-induced edema, indomethacin, or PTM extracts (100, 250, and 500 mg/kg). The effect of mature hydrophilic extract was further evaluated in an air pouch model, where rats received the placebo, carrageenan, indomethacin, or the extract (500 and 1000 mg/kg). Leukocytes, cytokines, and markers of oxidative stress were evaluated. The results showed the presence of organic compounds, total phenols, and flavonoids. The mature hydrophilic extract exhibited the highest antioxidant activity. At 500 mg/kg, it reduced edema, leukocyte migration, and levels of IL-1β, IL-6, and TNF-α while managing oxidative stress and preventing histological damage. In conclusion, PTM contains bioactive compounds with potential pharmacological properties. The hydrophilic extract of the mature pericarp, at a dose of 500 mg/kg, exhibits an enhanced antioxidant and anti-inflammatory effect.

## 1. Introduction

Inflammation is a biological response of tissue to injury, manifested in two phases: an acute phase, characterized by the presence of neutrophils and monocytes, and a chronic phase, characterized by the predominance of macrophages and lymphocytes [1]. Chronic diseases are linked to inflammation and oxidative stress, which cause cell damage [2]. Although conventional treatments such as non-steroidal anti-inflammatory drugs (NSAIDs) and corticosteroids are used to manage inflammation in diseases such as rheumatoid arthritis, these drugs cause adverse effects that affect other organs and systems, limiting the adherence to pharmacological treatments [3,4,5,6]. This situation highlights a knowledge gap and the need to develop safer and more effective drugs to regulate chronic inflammation in these cases [7].

Plant extracts serve as a valuable source of active compounds that are accessible to the general population, underscoring their importance in the search for complementary treatments. In this regard, conducting comprehensive phytochemical studies is crucial to identify the chemical components they contain and evaluate their pharmacological properties [8]. This process not only enhances our understanding of the mechanisms of action but also allows for the optimization of their clinical applications. Furthermore, their antioxidant and anti-inflammatory properties suggest potential roles in the prevention or complementation of treatments for diseases related to chronic inflammation [9]. The precise identification of these phytochemical components could lead to the development of more effective and safer therapies that tap into the rich tradition of natural medicine and the immense potential of biodiversity.

*Passiflora tripartita* var. *mollissima* is a plant species that produces aromatic fruits whose edible parts contain vitamins, minerals, carbohydrates, and flavonoids [10,11]. Traditionally, this fruit has been consumed by the population to alleviate constipation and high cholesterol [12]. Flavonoids in the form of C-glycosides are particularly abundant in the pericarp, the part of the fruit that is typically discarded, especially during its mature stage [13]. These compounds are widely recognized for their potent antioxidant and anti-inflammatory properties [14,15]. However, to date, no in vivo studies have confirmed the anti-inflammatory effects of the pericarp, and the bioactive components present in the green stage of the pericarp remain unidentified. Furthermore, it is essential to note that the pericarp of the Passiflora genus contains a variety of phenolic compounds and unsaturated fatty acids, which further contribute to its significant bioactive potential, making it a valuable resource for promoting health and wellness [16,17].

Therefore, a comprehensive phytochemical and antioxidant assessment of *P. tripartita* var. *Mollissima* extracts, obtained from the mature and green stages using hydrophilic and lipophilic solvents, is crucial to identify and confirm the plant’s pharmacological properties. This evaluation also serves to explore its potential applications in natural medicine. Furthermore, it is essential to assess its anti-inflammatory potential in vivo. For this purpose, models such as carrageenan-induced paw edema and the air pouch model in rodents are used, which allow precise measurement of both acute and chronic inflammation, facilitating complete integration and interpretation of the results [18]. In this context, the objective of this research is to evaluate the bioactive compounds, antioxidant capacity, and anti-inflammatory effects of the pericarp extracts of *P. tripartita* var. *mollissima* in two stages of ripening.

## 2. Results

### 2.1. Phytochemical Analysis

**Figure 1:**
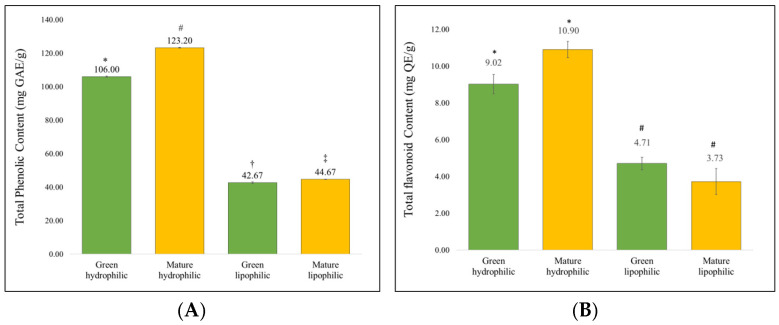
Total phenol content and Total flavonoid content. Data are presented as mean + SEM (*n* = 3). Panel (**A**) represents the gallic acid equivalent per gram of dry extract (GAE/g). Panel (**B**) represents the equivalent of quercetin per gram of dry extract (QE/g). Different symbols (*, #, †, ‡) indicate significant differences between groups (*p* < 0.05), as determined by Tukey’s post hoc test.

The components identified in the extracts by GC-MS are detailed in Table 1 and Table 2. The most abundant compounds were pentacosane and heptacosane in the green hydrophilic extract; α-d-Galactopyranose, l-glucose, heptacosane, and heptadecane in the green lipophilic extract; dl-Proline, 5-oxo-, methyl ester, nanocosane, and eicosane in the mature lipophilic extract; and tritetracontane, pentacosane, oleic acid, undecanoic acid, tetrapentacontane, and octadecane in the mature hydrophilic extract. Furthermore, Figure 1 shows the total content of phenol and flavonoids in the four extracts, measured by UV–VIS. The mature hydrophilic extract exhibited the highest levels of total phenols and flavonoids. The flavonoid content between mature and green hydrophilic extracts was comparable (*p* = 0.124).

### 2.2. Antioxidant Capacity

**Figure 2:**
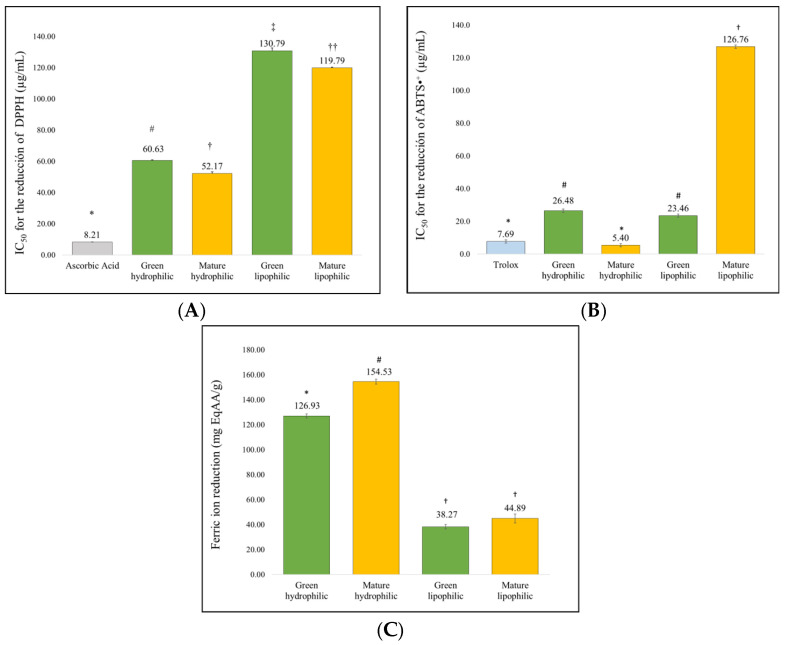
Reduction of free radicals and reduction of ferric ions by the extracts. Data are presented as mean + SEM (*n* = 3). Panels (**A**) and (**B**) represent the IC50 values for the radical scavenging activity of DPPH and ABTS•⁺ of different extracts. Panel (**C**) represents the capacity of the extracts to reduce ferric ions. Different symbols (*, #, †, ‡, ††) indicate significant differences between groups (*p* < 0.05), as determined by Tukey’s post hoc test.

Figure 2 compares the antioxidant capacity of the four types of extracts by evaluating their effectiveness against DPPH and ABTS•⁺ radicals, as well as their ability to reduce ferric ions. The mature hydroalcoholic extract exhibited significantly superior antioxidant capacity compared to the other extracts (*p* < 0.05). Furthermore, this reducing capacity was proportional to the concentration of the extract, as shown in Figure S1.

### 2.3. Anti-Inflammatory Effect on Plantar Edema

Table 3 shows that in the control group, the size of the edema on the rat paw progressively increased over the four hours of evaluation. Inflammation peaked in the fourth hour; however, no significant differences were observed compared to the third hour (*p* = 0.518). Oral administration of indomethacin, as well as hydrophilic and lipophilic extracts, prevented the increase in the size of the edema in the rat paw compared to the control group. However, it is important to note that this effect was not proportional to the concentration of extracts administered (Figure S2).

The mature and green hydrophilic extracts significantly reduced the size of the edema in the second hour (*p* < 0.05) compared to the control group. The mature hydrophilic extract at 500 mg/kg showed the greatest inhibition during the third and fourth hours, with 41.65% ± 2.30 and 39.47% ± 3.11, respectively.

Regarding the mature lipophilic extract, it significantly reduced the size of the edema in the first and fourth hours, while the green lipophilic extract achieved this in the second and fourth hours. In both cases, the reduction was significant (*p* < 0.05) compared to the control group. The mature lipophilic extract at 250 mg/kg showed inhibition of inflammation during the four hours, with reductions of 40.21% ± 1.49, 22.16% ± 1.34, 24.96% ± 9.35, and 30.96% ± 4.34, respectively. However, its efficacy did not exceed that of the mature hydrophilic extract at 500 mg/kg.

### 2.4. Anti-Inflammatory Effect on Carrageenan-Induced Air Pouches

Given that the hydrophilic extract at a dose of 500 mg/kg showed the most significant anti-inflammatory effect in the rat paw edema model, further evaluation was conducted using the carrageenan-induced air pouch model. The aim was to assess its impact on inflammatory parameters and oxidative stress.

### 2.5. Effect on the Number of Leukocytes in the Exudate

**Figure 3:**
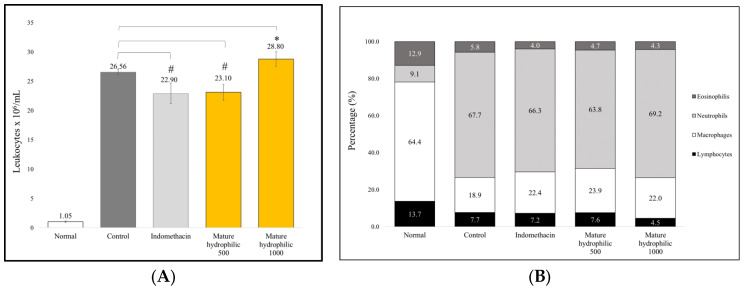
Number and differentiation of leukocytes in the exudate. Data are presented as mean + SEM (*n* = 6). Symbol (*) indicates a significant difference (*p* < 0.05) compared to the control group, while symbol (#) indicates that there is no significant difference (*p* > 0.05) compared to the control group, determined by Tukey’s post hoc test. (**A**) Number of leukocytes; (**B**) Leukocyte differentiation.

Figure 3A shows that the mature hydroalcoholic extract at 500 mg/kg reduced leukocyte migration, although the reduction was not statistically significant compared to the control group (*p* = 0.233). Figure 3B illustrates a decrease in the proportion of granulocytes and lymphocytes, along with an increase in macrophages, after treatment with extracts and indomethacin. Furthermore, Figure 3A shows that the extract at a dose of 1000 mg/kg did not reduce leukocyte migration.

### 2.6. Effect on Inflammatory Cytokines in the Exudate

Table 4 shows that in the group treated with carrageenan alone, the levels of IL-6, IL-1, and TNF-α increased significantly compared to the normal group (*p* < 0.05). In the groups that received mature hydroalcoholic extracts at 500 mg/kg and 1000 mg/kg, the level of IL-6 decreased, although this reduction was not significant (*p* > 0.05), with inhibitions of 6.61% ± 3.25 and 2.87% ± 2.22, respectively. On the contrary, the level of IL-1 was significantly reduced in the groups treated with indomethacin and with the extract at 500 mg/kg compared to the control group (*p* < 0.05), showing inhibitions of 63.76% ± 7.59 and 67.32% ± 10.83, respectively. Furthermore, the TNF-α level also decreased significantly in the groups treated with indomethacin and with the extract at 500 mg/kg compared to the control group (*p* < 0.05), with inhibitions of 27.96% ± 8.89 and 37.89% ± 2.84, respectively.

### 2.7. Effect on Oxidative Stress in the Exudate

Table 5 shows that in the group treated with carrageenan alone, there was a significant increase in the levels of MDA, GSH, and CAT (*p* < 0.05), while the NO levels decreased significantly (*p* < 0.05) compared to the normal group. Among the treated groups, the 500 mg/kg extract group demonstrated the greatest reduction in MDA levels, with a decrease of 10.19% ± 4.86; however, this reduction was not statistically significant (*p* > 0.05) compared to the control group. GSH levels in groups treated with 500 mg/kg and 1000 mg/kg extracts decreased significantly (*p* < 0.05) compared to the control group, with reductions of 7.88% ± 0.16 and 11.81% ± 0.06, respectively. NO levels were significantly higher (*p* < 0.05) in the treated groups compared to the control group. CAT levels increased in the 500 mg/kg extract group, but not significantly (*p* > 0.05), while the indomethacin group showed a significant increase (*p* < 0.05) compared to the control group.

### 2.8. Effect on Histological Alterations in Epithelial Tissue

In Table 6, the histological evaluation reveals differences in leukocyte infiltration and alterations in the thickness of the air pouch membrane between the treated and untreated groups. In the normal group, minimal leukocyte infiltration was observed in the dermis, hypodermis, and subcutaneous tissue, along with slight changes in the thickness of the air pouch membrane. On the contrary, the control group exhibited a moderate presence of erythrocytes in subcutaneous tissue, as well as significant infiltration of leukocytes and eosinophils at both the subcutaneous and muscular levels, resulting in a pronounced alteration in membrane thickness. The group treated with the extract at a dose of 500 mg/kg showed a significantly lower degree of infiltration and minimal alterations in the membrane; conversely, the group receiving 1000 mg/kg showed moderate leukocyte infiltration. Furthermore, the group treated with indomethacin demonstrated moderate infiltration and alterations in membrane thickness, comparable to those seen in the 1000 mg/kg group.

## 3. Discussion

In this study, for the first time, a GC-MS analysis was reported on the pericarp extracts of *Passiflora tripartita* var. *mollissima*. As shown in Table 1 and Table 2, the compounds identified in the highest amounts were heptacosane, heptadecane, dl-proline, 5-oxo-, methyl ester, eicosane, and oleic acid. These compounds have demonstrated antioxidant and anti-inflammatory properties [19,20,21,22,23]. Heptacosane acts as both a substrate and a potent P-glycoprotein (P-gp) inhibitor, demonstrating the ability to retain doxorubicin within cells and enhance its cytotoxic effects. Studies indicate that these compounds act as non-toxic modulators of P-gp through various mechanisms and can reverse resistance to P-gp-mediated drugs in tumor cells [19]. The second major compound, heptadecane, is a volatile compound that blocks fatty acid synthesis de novo and mitigates several stress-related diseases. Furthermore, it has been shown to exert anti-inflammatory effects in aged kidney tissue [20].

Figure 1 presents the results of the UV–VIS analysis, showing that the mature hydrophilic extract exhibited a higher content of total phenols and flavonoids (123.20 ± 0.23 mg GA/g and 10.90 ± 0.44 mg QE/g dry weight, respectively). Similarly, Simirgiotis et al. [13] also reported a higher amount of flavonoids (0.56 mg of quercetin/g of dry weight) in the same type of extract, although it was lower than the values observed in this study. Previous research on this species has focused on the glycosylated aromatic compounds of the fruit [24], cyanogenic glycosides [25], and their antihyperglycemic [26] and anti-inflammatory properties [27]. Furthermore, the plant is known to contain several key components, including alkaloids, saponins, flavonoids, triterpenoids, and proteins [28].

In the evaluation of the extracts against free radicals, Figure 2 shows that the mature hydrophilic extract exhibited a greater reducing capacity for DPPH, ABTS•⁺, and ferric ions. This activity could be related to the presence of polyphenols, oleic acid, and undecanoic acid identified in this study, all of which have previously demonstrated antioxidant capacity [23,29]. Additionally, the presence of C-glycoside flavonoids identified in the same type of extract by Simirgiotis et al. [13] could also contribute to this activity, as these compounds are known for their ability to reduce free radicals in vivo and in vitro [30].

The mature hydrophilic extract stands out as a source of bioactive compounds with antioxidant properties due to its high concentration of phenols, flavonoids, and fatty acids such as oleic and undecanoic acids. These components could have potential in the development of nutraceutical or pharmaceutical products for the prevention and treatment of diseases related to oxidative stress and inflammation. This research opens the opportunity to further explore the variability in the composition and biological activity of pericarp extracts based on their maturation to optimize their efficacy in future therapeutic applications.

The in vivo anti-inflammatory effect of the four types of extracts was evaluated. After subcutaneous administration of carrageenan to the plantar surface of the rats’ paws, a gradual increase in the thickness of the edema was observed over four hours, indicating inflammation (Table 3 and Figure S2). This inflammation is attributed to the ability of carrageenan to activate the complement system and trigger polymorphonuclear cell migration, as well as the release of cytokines, prostaglandins, and serotonin (5HT), which promotes vascularization in the affected area [31]. Following the administration of the extracts and indomethacin, a progressive and significant reduction (*p* < 0.05) in the thickness of the paw was observed over time. The mature hydrophilic extract at 500 mg/kg showed the greatest reduction in the size of the edema compared to the other extracts, with its effect beginning at the second hour and lasting until the fourth hour, at which point it began to decrease. On the contrary, indomethacin showed a growing effect even at the fourth hour; however, when comparing the thickness of the paw between the two, no significant differences were observed (*p* < 0.05). The effect of the mature hydrophilic extract could be related to its antioxidant capacity, as reported in this study, as well as previous research that demonstrated that metabolites such as oleic acid and C-glycoside flavonoids present in this extract have anti-inflammatory properties [23,30].

The carrageenan-induced air pouch inflammation model was used to further explore the effect of the mature hydrophilic extract on biochemical markers associated with inflammation. In the control group, a significant increase in exudate volume and leukocyte count was observed (*p* < 0.05), along with an elevated neutrophil proportion compared to the normal group (Figure 3 and Figure S3). Additionally, a significant increase in IL-6, IL-1β, and TNF-α was recorded (Table 4). This effect is due to carrageenan activating the NF-κB pathway through toll-like receptor 4, inducing the transcription of pro-inflammatory cytokines [32,33,34]. This leads to increased vascular permeability and chemotaxis, which attract neutrophils and monocytes [35,36]. Excessive production of superoxide (O_2_^−^) by neutrophils can prolong inflammatory damage [37,38]. The interaction between NO and O_2_⁻ generates peroxynitrite (ONOO^−^), which exacerbates oxidative damage [39]. In Table 5, a significant decrease in NO (*p* < 0.05) is observed in the control group, likely due to its consumption in ONOO⁻ production [40]. In addition, there was a significant increase in MDA, a marker of oxidative stress [41]. As a compensatory response, GSH levels and CAT activity increased, although these mechanisms might be insufficient in cases of severe oxidative stress [42,43].

Our findings indicate that the mature hydrophilic extract moderately reduces leukocyte migration and alters the cellular composition at the inflammatory site, as well as significantly influencing some key inflammatory mediators, though with certain limitations. Regarding leukocyte migration, Figure 3A suggests that the 500 mg/kg extract has an initial anti-inflammatory effect, but it is not powerful enough to achieve statistical significance in reducing migrating leukocytes at the evaluated dose. Additionally, Figure 3B shows that the extract decreased the proportion of granulocytes and lymphocytes while increasing the proportion of macrophages at the inflammatory site. This change in cellular composition suggests that the extract may promote inflammation resolution, as macrophages are crucial in this phase due to their role in phagocytosis and tissue repair [44]. However, the 1000 mg/kg extract did not show a further improvement in reducing leukocyte migration, indicating that increasing the dose does not enhance this effect.

Regarding cytokines, Table 4 shows that in the groups treated with the extract at 500 mg/kg and 1000 mg/kg, the levels of IL-6 decreased, although this reduction was not significant (*p* > 0.05). The observed inhibition of 6.61% ± 3.25 and 2.87% ± 2.22, respectively, suggests a trend toward reducing IL-6, but it is not strong enough to be conclusive at the evaluated doses. On the other hand, IL-1 and TNF-α levels were significantly reduced (*p* < 0.05) in the groups treated with indomethacin and the 500 mg/kg extract. The inhibition of IL-1 was 63.76% ± 7.59 and 67.32% ± 10.83, respectively, while for TNF-α, it was 27.96% ± 8.89 and 37.89% ± 2.84, respectively. These results highlight the effectiveness of the 500 mg/kg extract in reducing these inflammatory mediators, consistent with previous studies documenting the ability of hydroalcoholic extracts of the *Passiflora* genus to modulate IL-1 and TNF-α [45,46], as well as secondary metabolites identified in the mature hydrophilic extract such as schaftoside and orientin, both C-glycoside flavonoids. These compounds have been documented for their ability to reduce inflammation by inhibiting the TLR4/MyD88 and TLR4/NF-κB pathways, resulting in a decrease in the release of IL-1, TNF-α, and IL-6 [14,15].

Regarding the evaluation of antioxidants in the exudate of the inflamed area, Table 5 and Figure S5 show that only the group treated with the 500 mg/kg extract exhibited a reduction in MDA of 10.19% ± 4.86, although it was not statistically significant (*p* = 0.362), suggesting a trend toward mitigating oxidative damage. However, this is not conclusive. Concerning GSH levels, the groups treated with extracts at 500 mg/kg and 1000 mg/kg showed reductions of 7.88% ± 0.16 and 11.81% ± 0.06, respectively, which were statistically significant (*p* < 0.05) compared to the control group. This decrease might indicate that the extract promotes the use of GSH to counteract inflammation-induced oxidative stress, hence the reduction in GSH levels. This could be due to the fact that flavonoids of *Passiflora* species are capable of increasing glutathione peroxidase activity during inflammation, thus reducing GSH levels [47]. Regarding NO, the levels in the extract-treated groups were significantly higher (*p* < 0.05) compared to the control group. This increase may reflect an adaptive response to improve vasodilation and perfusion at the inflamed site, which has been reported for *Passiflora* species in a review article [48], thus facilitating the resolution of inflammation. Regarding CAT, the 500 mg/kg extract-treated group showed an increase of 18.18% ± 8.21, which is not statistically significant compared to the control group (*p* = 0.149), suggesting an improvement in antioxidant capacity that is not conclusive.

Regarding the histopathological evaluation in Table 6, the normal group showed minimal infiltration of leukocytes and slight alteration in the thickness of the air pouch membrane, representing a basal state with no significant inflammation. On the contrary, the control group exhibited significant leukocyte infiltration and intense membrane alteration, indicating a robust inflammatory response in the absence of treatment. The indomethacin-treated group showed moderate leukocyte infiltration and membrane alteration. On the other hand, the groups treated with the extract, both at 500 mg/kg and 1000 mg/kg doses, demonstrated a reduction in inflammation, with less pronounced leukocyte infiltration and membrane alteration compared to the control group, suggesting that the extract has anti-inflammatory properties.

The hydroalcoholic extract of mature pericarp shows therapeutic potential as an anti-inflammatory agent, demonstrating a moderate ability to reduce leukocyte migration and alter the cellular composition at the inflammatory site, thus promoting the resolution of inflammation through an increase in the proportion of macrophages. At a dose of 500 mg/kg, the extract significantly reduced IL-1 and TNF-α, though its effect on IL-6 was limited. The presence of flavonoids such as schaftoside and orientin, along with fatty acids such as oleic and undecanoic acid, highlights the importance of understanding the phytochemical profile of the extract to optimize its use. Although some changes in oxidative stress markers were not significant, the reduction in MDA and GSH, as well as the increase in CAT and NO, suggests antioxidant properties and a regulatory effect on inflammation and vascular function. These results underscore the need for further studies to confirm the safety and efficacy of the extract in chronic anti-inflammatory treatments.

The limitations of this research are as follows. Although the quantification of components was performed using GC-MS, additional analysis with liquid chromatography would be necessary to identify non-volatile components. Furthermore, while the pharmacological models used allowed for biochemical and histopathological evaluation of the hydrophilic extract of mature *Passiflora tripartita* pericarp, the precise mechanism by which it regulates the inflammatory process remains unexplained, requiring further molecular studies. Additionally, the findings are limited to animal models, so human studies are needed to clinically extrapolate the results. Finally, the lack of improvement in effects at higher doses suggests an efficacy threshold at moderate doses, highlighting the need to optimize dosing and better understand the mechanisms of action.

## 4. Materials and Methods

### 4.1. Reagents and Equipment

Aluminium trichloride (AlCl_3_), Folin–Ciocalteu reagent, 2,2-difenil-1-picrilhidracilo (DPPH), 2,2′-azinobis(3-etilbenzotiazolin-6-sulfónico) (ABTS), Ferric chloride hexahydrate (FeCl_3_·6H_2_O), Ascorbic acid (AA), Carrageenan type IV, thiobarbituric acid (TBA), potassium hexacyanoferrate III, and Trichloroacetic acid (TCA) were purchased from Sigma-Aldrich, St. Louis, MO, USA. Spectrophotometer UV-VIS GENESYS^®^ 10S, Waltham, MA, USA; analytical balance 0.0001 g OHAUS CORPORATION, Parsippany, NJ, USA; microscope LEICA DM 750 with camera LEICA ICC50 W, Heerbrugg, Switzerland; TRACE^TM^ 1310 gas chromatograph, Waltham, MA, USA, coupled to an ISQ7000 mass spectrometer, Bartlesville, Oklahoma, United States; Digimatic micrometer 0.001 mm Mitutoyo Corporation, Kawasaki, Japan.

### 4.2. Research Design

An experimental design was conducted in which the intervention was considered the independent variable and its effects were the dependent variable, measured quantitatively. The working groups were randomly formed and the results of the intervention groups were compared with those of the control group.

### 4.3. Study Population

To evaluate inflammation in the paw edema and air pouch models, 84 and 30 male Holtzman rats (weighing 210 ± 10 g), respectively, obtained from the National Institute of Health Biotherium were used. The rats were randomly grouped into 6 rats per group.

### 4.4. Handling of Experimental Animals

The experiment was carried out following the guidelines of the “Guide for the Care and Use of Laboratory Animals” published by the National Research Council of the United States [49]. The housing environment allowed for the fulfillment of the five freedoms. The ambient temperature was maintained at 22 ± 2 °C, humidity at 55 ± 10%, and noise level at <20 kHz. The animals had access to 12 h light–dark cycles and received a balanced rodent diet from the Universidad Agraria la Molina and purified water ad libitum.

The research protocol was reviewed and approved by the Ethics Committee of the Faculty of Pharmacy and Biochemistry of the Universidad Nacional Mayor de San Marcos on 7 November 2023 (Certificate number 012-CE-UDI-FFB-2023).

### 4.5. Plant Species

The plant species (branch and fruit) were collected in the village of Raquina in the Pucará district of Huancayo province in Perú. The sample was then deposited in the Herbarium of the Natural History Museum at the National University of San Marcos, where it was classified as *Passiflora tripartita* (Juss.) Poir. *Var. mollissima* (Kunth) Holm-Niels. & P. Jorg. (Certificate No. 014-USM-2021). The fruits were grouped into green and mature stages and then the pericarps were separated from the seeds.

### 4.6. Extraction of Chemical Components

The extraction of bioactive compounds was performed according to Zucolotto et al. [50]. The pericarps were washed with potable water, dried in the shade for 1 day, and then dried in an oven at 40 °C ± 2 for 2 days and ground with an electric mill to obtain 3 mm particles. Lipophilic and hydrophilic extracts were obtained by maceration with n-hexane and 70° ethanol, respectively, in a 1:10 ratio for 7 days. After recovering the solvent with a rotary evaporator and drying in an oven at 40 °C, the moisture content of the extracts was determined by gravimetry. For this purpose, 1 g of each extract was weighed and dried in an oven at a temperature of 100 °C to 105 °C for 2 h. After the samples had cooled, they were weighed again. The percentage of moisture was calculated on the basis of the recorded weight difference. Four extracts were obtained: mature hydrophilic, mature lipophilic, green hydrophilic, and green lipophilic, with a moisture of 2.5%, 1.8%, 2.7%, and 1.4%, respectively.

### 4.7. Chemical Analysis

Chemical constituents were identified using a GC-MS system. A total of 10 mg of hydroalcoholic and lipophilic extracts were diluted in 1 mL of methanol and dichloromethane, respectively, and then 1 µL of each diluted solution was injected to determine volatile compounds. The GC-MS used a DB-5MS column (30 m × 250 µm × 0.25 µm) with a temperature program starting at 50 °C, held for 5 min, followed by an increase of 3 °C/min to 155 °C, and then a further increase of 15 °C/min to 250 °C, which was held for 2 min. The injection system was set to operate in split mode (40:1), with helium as the carrier gas at a flow rate of 1 mL/min [51]. Identification of secondary metabolites was performed by comparison of retention time (Rt) and mass spectral data with the NIST20 library and the relevant literature [52]. The relative area of each compound in the extract was expressed as a percentage, which was determined by comparing the area of each chromatogram peak with the total area of all identified peaks. No correction factors were applied.

### 4.8. Total Phenol Content

Phenols were quantified by reducing the Folin–Ciocalteu reagent [53]. Twenty-five milligrams of extract were weighed and dissolved in methanol at a concentration of 100 µg/mL. From this solution, 500 µL were taken and 250 µL of Folin–Ciocalteu reagent was added. After 5 min, 1250 µL of a 20% sodium carbonate solution was added to alkalize the sample. After a 2 h incubation protected from light, the absorbance was measured on a UV–VIS spectrophotometer at 760 nm. Three samples were analyzed for each extract. To calculate the phenol concentration in the samples, a calibration curve prepared with standard gallic acid solutions was used. The results were expressed in milligrams of equivalent gallic acid per gram of dry extract weight (mg GAE/g).

### 4.9. Total Flavonoid Content

Total flavonoids were determined by reducing AlCl_3_ [54]. Twenty-five milligrams of extract were diluted with methanol at a concentration of 100 µg/mL. To 0.5 mL of the sample, 2.25 mL of H_2_O and 0.15 mL of 5% NaNO_2_ were added. After 6 min, 10% AlCl_3_·H_2_O was added, the mixture was incubated for 5 min at room temperature, and then 1 mL of 1M NaOH was added. The mixture was vortexed, and the absorbance was measured using a UV–VIS spectrophotometer at 510 nm. Three samples were analyzed for each extract. To calculate the total flavonoid content in the samples, we used a calibration curve prepared with standard quercetin solutions. The results were expressed in milligrams of quercetin equivalent per gram of dry extract weight (mg QE/g).

### 4.10. Ability to Reduce 2,2-Diphenyl-1-Picrylhydrazyl (DPPH)

The reduction of the DPPH radical was analyzed by measuring the decrease in violet color, evaluated with the absorbance value according to the Prieto method [55]. A 0.4 mmol DPPH radical solution was prepared in HPLC-grade methanol. The extracts were diluted into five concentrations with final volumes of 100 µL in a microplate, to which 100 µL of the DPPH solution was added. A group receiving only the DPPH radical solution (control) and a group receiving only the solvent (blank) were included. The samples were then incubated at 25 °C for 30 min, after which the absorbance was measured at 517 nm using a microplate reader. To compare the reduction capacity of the extracts, the inhibitory concentration value 50 (IC50), expressed in µg/mL, was used.

### 4.11. Ability to Reduce 2,2′-Azino-Bis(3-Ethylbenzothiazoline-6-Sulfonic Acid) (ABTS•⁺)

This was assessed using the ABTS•⁺ radical decolorization technique, following the method described by Re et al. [56]. The ABTS•⁺ radical was generated by reacting ABTS (7 mM) with potassium persulfate (2.45 mM) in the dark for 16 h at room temperature. The solution was diluted to achieve an absorbance of 0.700 ± 0.020 at 734 nm. The four extracts were tested at five concentrations by mixing 100 µL of each sample with 1 mL of the ABTS•⁺ solution and incubating in the dark for 30 min. Absorbance was then measured at 734 nm using a UV–VIS spectrophotometer. The results were expressed as the inhibitory concentration 50 (IC50) and reported in µg/mL.

### 4.12. Ferric Reducing Antioxidant Power (FRAP)

This assay followed the method described by Belyagoubi et al. [57] that measures the reduction of ferric iron (Fe^3+^) to ferrous ion (Fe^2+^), resulting in a change of color from light blue to deep blue. The FRAP reagent was freshly prepared using acetic acid–sodium acetate buffer (pH 3.6), TPTZ, and FeCl_3_ in a 25:2.5:2.5 mL ratio, then diluted to 80 mL with deionized water. A calibration curve with ascorbic acid (AA) was created at concentrations of 1, 2, 3, 4, 5, and 10 ppm, requiring a correlation coefficient between 0.8 and 1. Three samples of each extract were prepared at 100 ppm. From the latter, 0.5 mL was mixed with 1.5 mL of FRAP reagent, giving a final concentration of 10 ppm. After 60 min in the dark, the absorbance at 593 nm was measured using a UV–VIS spectrophotometer. The results were expressed as milligrams of ascorbic acid equivalent per gram of dry extract (mg EAA/g).

### 4.13. Effect on Inflammation on the Plantar Surface of Rats

Inflammation was induced with 100 μL of 1.5% carrageenan on the plantar surface of the right hind paw. Subsequently, placebo (2 mL/kg), indomethacin, or extracts were administered orally to the formed groups. Paw thickness was measured with a micrometer before inflammation induction and one, two, three, and four hours after treatment [58]. The groups were as follows: I: Control (Carrageenan), II: Indomethacin 5 mg/kg, III: Green hydrophilic 100 mg/kg, IV: Green hydrophilic 250 mg/kg, V: Green Hydrophilic 500 mg/kg, VI: Hydrophilic mature 100 mg/kg, VII: Hydrophilic mature 250 mg/kg, VIII: Hydrophilic mature 500 mg/kg, IX: Green lipophilic 100 mg/kg, X: Green lipophilic 250 mg/kg, XI: Green lipophilic 500 mg/kg, XII: Mature lipophilic 100 mg/kg, XIII: Mature lipophilic 250 mg/kg, and XIV: Mature lipophilic 500 mg/kg.

### 4.14. Effect on Inflammation in the Air Pouch Model with Carrageenan

#### 4.14.1. Induction of Inflammation and Treatments

The hydrophilic fraction of the mature pericarp was evaluated in an air pouch model in rats [59]. Groups of 6 rats were formed: I: Normal, II: Control (Carrageenan), III: Indomethacin 5 mg/kg + carrageenan, IV: mature hydrophilic 500 mg/kg + carrageenan, and V: mature gydrophilic 1000 mg/kg + carrageenan.

The first day after shaving the rats’ backs, 10 mL of sterile air was administered subcutaneously. On the third day, 5 mL of air was administered in the same area, and on the sixth day, the corresponding groups received oral treatment. Three hours after treatment, the groups who received treatment received 2 mL of 2% carrageenan solution subcutaneously into the air pouch. After twelve hours, rats were sacrificed with pentobarbital (50 mg/kg) intraperitoneally, followed by exsanguination as described by Tobar et al. [60]. A total of 5 mL of wash solution was administered to the air pouch and a gentle massage was applied for homogenization. A sagittal cut of approximately 2 cm was made and the exudate was collected with a transfer pipette into chilled conical tubes, which were then centrifuged at 3000 rpm. The supernatant was separated for biochemical and immunological analyses and the pellet was used for leukocyte counting. Finally, air pouch skin samples measuring approximately 2 cm by 0.5 cm were taken and fixed in 10% buffered formalin (*v*/*v*).

#### 4.14.2. Number and Differentiation of Leukocytes

To 0.5 mL of the previously obtained pellet, 1.5 mL of chilled PBS was resuspended. Leukocyte counting was performed using a Neubauer chamber after Giemsa staining, and differential cell counting was performed using Wright–Giemsa staining [59].

#### 4.14.3. Inflammatory Cytokines

This was carried out using a sandwich ELISA method in exudate with specific Sigma-Aldrich rat kits, USA, to determine the concentrations of IL-6, IL-1β, and TNF-α. Samples and standards were prepared and added to plates coated with capture antibodies. After incubation and washing, a conjugated enzyme-conjugated detector antibody was added, followed by further incubation and washing. The enzyme substrate was added, and the reaction was stopped with a stop solution. Absorbance was measured using a microplate reader at 450 nm, and concentrations were calculated using a standard curve.

#### 4.14.4. Nitric Oxide

Nitric oxide (NO) was evaluated in the exudate. It was determined by reducing the nitrates using Griess reagent [61]. First, 800 µL of the sample, 700 µL of H_2_O, and 100 µL of 1M NaOH were added. After 5 min, 100 µL of 30% ZnSO_4_ was added. The mixture was stirred until a milky suspension was obtained and centrifuged at 3000 rpm for 15 min. To 920 µL of the obtained supernatant, a zinc powder aliquot was added, stirred, and left to stand for 45 min. Then, it was centrifuged again at 3000 rpm for 15 min and 800 µL of supernatant and 800 µL of a mixture of 2% sulfanilamide with 0.1% N-1-naphthylethylenediamine were added. The absorbance was determined with a UV–VIS spectrophotometer at 540 nm. Six samples were analyzed. To calculate the NO content in the samples, a calibration curve prepared with sodium nitrite standard was used. The results were expressed in µmol/g.

#### 4.14.5. Glutathione

Glutathione (GSH) was evaluated in the exudate. It was determined by the reaction with DTNB [62]. To 0.5 mL of the sample, 1.5 mL of 0.02 M TRIS buffer pH 8.2 and 0.1 mL of 5.5 1-Dithiobis (2-nitrobenzoic acid) 0.01 M were added, then the volume increased to 10 mL with absolute methanol. A blank without a sample and a blank without DTNB were prepared, then the tubes were capped with a rubber stopper and allowed to stand for 15 min, after which they were centrifuged at 3000 rpm for 15 min. The absorbance was determined at 412 nm on a UV–VIS spectrophotometer. Six samples were analyzed. The GSH content was determined using the molar extinction coefficient of 13,100 M^−1^·cm^−1^. The results were expressed as nanomoles per gram (nmol/g).

#### 4.14.6. Catalase

Catalase (CAT) activity was determined in the exudate. It was determined by the reduction of dichromate/acetic acid to chromic acetate [63]. To 100 µL of supernatant, 2.9 mL of 0.05% H_2_O_2_ was added. The absorbance was immediately measured with a UV–VIS spectrophotometer at 240 nm every 15 s for 2 min. The reaction was stopped by adding 2 mL of dichromate-acetic acid reagent. Six samples were analyzed. CAT activity was determined by dividing the absorbance difference per minute by the milligrams of protein per milliliter and then multiplying by 7500. The results were expressed in units per milligram (U/mg) of protein.

#### 4.14.7. Malondialdehyde

Malondialdehyde (MDA) was determined in the exudate. It was determined by reaction with thiobarbituric acid [64]. First, 0.5 mL of the sample was mixed with 1 mL of 20% trichloroacetic acid, then after stirring, it was centrifuged for 10 min at 3000 rpm. To 1 mL of the supernatant, 1.5 mL of 0.67% thiobarbituric acid in 0.25 N HCl was added, stirred, and placed in a boiling water bath for 30 min. Then, it was cooled with ice water. The absorbance was determined with a UV–VIS spectrophotometer at 535 nm. Six samples were analyzed. The concentration of MDA in the samples was calculated using an extinction coefficient of 156,000 M^−1^·cm^−1^. The result was expressed as nanomoles per gram (nmol/g).

#### 4.14.8. Histopathological Study

Histopathological slides were prepared from skin sections as described by Kim et al. [65]. The skin sections were dehydrated with ethanol and embedded in paraffin. From this, 5 µm thick sections were obtained and stained with hematoxylin and eosin. Morphological evaluation was performed using a light microscope, focusing on leukocyte infiltration in the dermis, subcutaneous tissue, muscle layer, and air pouch membrane. Additionally, the thickness and number of lymphocytes per field in the air pouch membrane were assessed.

#### 4.14.9. Data Processing and Analysis

Results for polyphenol content, flavonoid content, antioxidant capacity, edema size, and biochemical parameters of the exudate were presented as means plus/minus the standard error of the mean (SEM). The homogeneity of the variance was determined using the Levene test and normality was assessed with the Shapiro–Wilk test. Then, ANOVA was performed to check for variance and, finally, Tukey’s test was conducted to evaluate differences between groups. All analyses were assessed at a significance level of *p* < 0.05.

## 5. Conclusions

The pericarp of *Passiflora tripartita* var. *mollissima* contains organic compounds with pharmacological potential, including heptacosane, heptadecane, dl-proline, eicosane, oleic acid, total phenols, and flavonoids. The hydrophilic extract derived from mature pericarp demonstrated an enhanced antioxidant capacity through the reduction of free radicals. At a dose of 500 mg/kg, it has an anti-inflammatory profile, as evidenced by a reduction in paw edema in rats, inhibition of leukocyte infiltration, regulation of oxidative stress, modulation of cytokines such as IL-1β, IL-6, and TNF-α in inflammatory exudate, and decreased membrane disruption and leukocyte migration, as observed in histopathological analysis of skin biopsies. These effects suggest a beneficial role in resolving inflammation and mitigating oxidative stress caused by carrageenan-induced alterations. These findings underscore the therapeutic potential of the extract due to its antioxidant and anti-inflammatory properties, offering promising opportunities for its application in the development of nutraceutical and pharmaceutical products aimed at treating diseases related to inflammatory and oxidative stress.

## Figures and Tables

**Table 1:** Bioactive compounds of the green pericarp of *Passiflora tripartita* var. *mollissima* fruit.

	N°	Compound	Formula	Molecular Weight	CAS Number	Rt (min)	Ra (%)
Green hydrophilic extract	1	α-d-Galactopyranose	C_6_H_12_O_6_	180.0800	26566-61-0	15.76	1.26
	2	Isoglutamine	C_5_H_10_N_2_O_3_	146.1460	636-65-7	22.79	1.02
	3	Pentacosane	C_25_H_52_	352.6880	629-99-2	44.55	14.46
	4	Heptacosane	C_27_H_56_	380.7420	593-49-7	39.06	81.26
	5	Imidazole	C_3_H_4_N_2_	68.0782	288-32-4	39.47	0.97
	6	Glucopyranuramide	C_7_H_14_O_6_	194.1850	97-30-3	42.47	1.03
		Total components					100.00
Green lipophilic extract	1	3-methylbutyl-oxirane	C_7_H_14_O	114.1880	5063-65-0	12.50	7.53
	2	α-d-Galactopyranose	C_6_H_12_O_6_	180.0800	26566-61-0	15.75	11.64
	3	Methylene asparagine	C_5_H_8_N_2_O_3_	144.1300	64414-81-9	15.90	6.76
	4	l-glucose	C_6_H_12_O_6_	180.1580	921-60-8	24.07	18.94
	6	Heptacosane	C_27_H_56_	380.7420	593-49-7	39.06	31.08
	7	Hexadecane	C_12_H_26_C_4_H_8_	226.4460	544-76-3	39.46	2.91
	5	Imidazole	C_3_H_4_N_2_	68.0782	288-32-4	39.47	5.49
	8	Heptadecane	C_17_H_36_	240.4730	629-78-7	41.57	15.65
		Total components					100.00

Rt, retention time; Ra, relative area.

**Table 2:** Bioactive compounds of the mature pericarp of *Passiflora tripartita* var. *mollissima* fruit.

	N°	Compound	Formula	Molecular Weight	CAS Number	Rt (min)	Ra (%)
Mature lipophilic extract	1	Acetamide	CH_5_CON	59.0678	60-35-5	4.22	3.00
	2	9-Hexadecenoic acid	C_16_H_30_O_2_	254.4130	373-49-9	4.93	1.49
	3	2-Butenoic acid	C_4_H_6_O_2_	86.0904	3724-65-0	6.72	5.40
	4	Isoserine	C_3_H_7_NO_3_	105.093	632-11-1	7.51	1.80
	5	Glycine	C_2_H_5_NO_2_	75.0672	56-40-6	12.53	1.20
	6	3-Acetylthymine	C_7_H_8_N_2_O_3_	168.1520	958996-65-1	13.77	2.50
	7	α-d-Galactopyranose	C_6_H_12_O_6_	180.0800	26566-61-0	15.80	6.16
	8	Methylene asparagine	C_5_H_8_N_2_O_3_	144.1300	64414-81-9	15.89	0.95
	9	dl-Proline, 5-oxo-, methyl ester	C_6_H_9_NO_3_	143.1406	54571-66-3	22.91	21.55
	10	Isoglutamine	C_5_H_10_N_2_O_3_	146.1460	636-65-7	24.46	2.35
	11	Caffeine	C_8_H_10_N_4_O_2_	194.1930	58-08-2	33.84	3.42
	12	2,6,10-Trimethyltetradecane	C_17_H_36_	240.4730	14905-56-7	33.93	0.24
	13	Pentadecanoic acid	C_15_H_30_O_2_	242.4020	1002-84-2	34.61	3.76
	14	Hexadecanoic acid, methyl ester	C_17_H_34_O_2_	270.4560	112-39-0	35.48	2.38
	15	Heptadecanoic acid, methyl ester	C_18_H_36_O_2_	284.4830	1731-92-6	35.90	2.65
	16	Methyl 9-*cis*,11-*trans*-octadecadienoate	C_19_H_34_O_2_	294.4780	13058-52-1	37.85	3.73
	17	Methyl (10*E*)-10-octadecenoate	C_19_H_36_O_2_	296.4940	13481-95-3	37.95	5.61
	18	Nonadecanoic acid	C_19_H_38_O_2_	298.5100	646-30-0	38.39	1.65
	19	Nonacosane	C_29_H_60_	408.7950	630-03-5	39.04	16.32
	20	Eicosane	C_20_H_42_	282.5530	112-95-8	40.12	13.84
		Total components					100.00
Mature hydrophilic extract	1	α-d-Galactopyranose	C_6_H_12_O_6_	180.0800	26566-61-0	15.77	1.18
	2	Isoglutamine	C_5_H_10_N_2_O_3_	146.1460	636-65-7	22.87	1.98
	3	Undecanoic acid	C_11_H_22_O_2_	186.2940	112-37-8	34.91	2.37
	4	*N*-hexadecanoic acid	C_16_H_32_O_2_	256.4290	57-10-3	35.45	2.25
	5	2,6,10-Trimethyltetradecane	C_17_H_36_	240.4730	14905-56-7	37.42	1.86
	6	6-Hydroxy-9-[tetrahydro-2*H*-pyran-2-yl]-9*H*-purine	C_10_H_12_N_4_O_2_	220.2310	446832-09-3	39.38	8.78
	7	9,10-Secocholesta-5,7,10(19)-triene-3,24,25-triol	C_27_H_44_O_3_	416.6450	272776-87-1	39.66	0.59
	8	Tritetracontane	C_43_H_88_	605.1720	7098-21-7	40.09	32.38
	9	3-*cis*, 9-*cis*, 12-*cis*-octadecatrienoate	C_19_H_32_O_2_	292.5000	75315-95-6	40.52	3.29
	10	Myristoleic acid	C_14_H_26_O_2_	226.3590	544-64-9	41.73	1.30
	11	Pentacosane	C_25_H_52_	352.6880	629-99-2	44.57	21.21
	12	Ethyl iso-allocholate	C_26_H_44_O_5_	436.6320	47676-48-2	46.18	2.03
	13	Oleic acid	C_18_H_34_O_2_	282.4670	112-80-1	47.42	3.88
	14	9-octadecenoic acid	C_18_H_34_O_2_	282.5000	2027-47-6	48.18	2.57
	15	Octadecane	C_18_H_38_	254.5000	593-45-3	48.83	4.48
	16	Tetrapentacontane	C_54_H_110_	759.4670	5856-66-6	49.45	3.86
	17	Docosanoic acid	C_22_H_44_O_2_	340.5900	112-85-6	50.22	2.08
	18	Sarreroside	C_30_H_42_O_10_	562.6000	545-36-8	52.85	1.96
	19	β-Tocopherol	C_28_H_48_O_2_	416.7000	16698-35-4	53.67	1.95
		Total components					100.00

Rt, retention time; Ra, relative area.

**Table 3:** Inhibition of edema size and inflammation in the rat paw.

Group (n)	1 h		2 h		3 h		4 h	
	Edema Size (mm)	Inhibition (%)	Edema Size (mm)	Inhibition (%)	Edema Size (mm)	Inhibition (%)	Edema Size (mm)	Inhibition (%)
Control	1.62 ± 0.06		1.79 ± 0.04		2.06 ± 0.04		2.15 ± 0.04	
Indomethacin	1.16 ± 0.15 #	28.45 ± 9.41	1.09 ± 0.14 *	39.29 ± 7.66	1.13 ± 0.06 *	44.89 ± 3.09	0.55 ± 0.15 *	74.46 ± 7.09
Green hydrophilic 500	1.23 ± 0.01 #	23.71 ± 0.90	1.27 ± 0.13 *	29.24 ± 7.46	1.26 ± 0.04 *	38.90 ± 2.03	1.36 ± 0.04 *	37.00 ± 1.90
Green hydrophilic 250	1.39 ± 0.08 #	14.02 ± 4.68	1.61 ± 0.06 #	9.87 ± 3.48	1.85 ± 0.08 #	10.05 ± 3.71	1.65 ± 0.06 *	23.22 ± 2.95
Green hydrophilic 100	1.18 ± 0.20 #	27.01 ± 12.13	1.52 ± 0.13 #	15.08 ± 7.39	1.57 ± 0.13 #	23.82 ± 6.37	1.59 ± 0.08 *	26.01 ± 3.50
Mature hydrophilic 500	1.18 ± 0.06 #	27.22 ± 3.67	1.23 ± 0.08 *	31.47 ± 4.68	1.20 ± 0.05 *	41.65 ± 2.30	1.30 ± 0.07 *	39.47 ± 3.11
Mature hydrophilic 250	1.41 ± 0.02 #	12.58 ± 1.03	1.47 ± 0.02 #	17.88 ± 0.85	1.82 ± 0.15 #	11.51 ± 7.17	1.95 ± 0.04 #	9.44 ± 1.93
Mature hydrophilic 100	1.56 ± 0.02 #	3.71 ± 1.25	1.41 ± 0.01 #	21.04 ± 0.49	1.98 ± 0.17 #	3.89 ± 8.22	1.80 ± 0.21 #	16.41 ± 9.84
Green lipophilic 500	1.17 ± 0.08 #	27.63 ± 4.80	1.67 ± 0.02 #	6.70 ± 1.16	1.65 ± 0.23 #	19.61 ± 11.17	1.60 ± 0.02 *	25.70 ± 1.07
Green lipophilic 250	1.22 ± 0.05 #	24.33 ± 2.97	1.35 ± 0.08 *	24.77 ± 4.52	1.51 ± 0.15 #	26.74 ± 7.33	1.51 ± 0.09 *	29.72 ± 4.10
Green lipophilic 100	1.58 ± 0.20 #	2.47 ± 12.32	1.37 ± 0.07 *	23.28 ± 3.78	1.56 ± 0.13 #	23.99 ± 6.54	1.53 ± 0.08 *	28.79 ± 3.66
Mature lipophilic 500	1.21 ± 0.05 #	25.15 ± 3.17	1.43 ± 0.06 #	19.93 ± 3.42	1.82 ± 0.05 #	11.35 ± 2.35	1.67 ± 0.06 *	22.60 ± 2.63
Mature lipophilic 250	0.97 ± 0.02 *	40.21 ± 1.49	1.39 ± 0.02 #	22.16 ± 1.34	1.54 ± 0.19 #	24.96 ± 9.35	1.49 ± 0.09 *	30.96 ± 4.34
Mature lipophilic 100	0.98 ± 0.02 *	39.18 ± 1.44	1.56 ± 0.03 #	13.04 ± 1.83	2.04 ± 0.07 #	0.65 ± 3.38	1.56 ± 0.06 *	27.71 ± 2.65

Each column represents the mean + SEM (*n* = 6). Symbol (*) indicates a significant difference (*p* < 0.05) compared to the control group, while symbol (#) indicates that there is no significant difference (*p* > 0.05) compared to the control group, determined by Tukey’s post hoc test.

**Table 4:** Inflammatory cytokines in exudate.

Group (n)	IL-6		IL-1 β		FNT-α	
	pg/mL	Inhibition (%)	pg/mL	Inhibition (%)	pg/mL	Inhibition (%)
Normal	214.51 ± 7.01 *	-	115.45 ± 4.74 *	-	1.96 ± 0.48 *	-
Control	1443.10 ± 30.72	-	631.88 ± 21.29	-	55.30 ± 2.91	-
Indomethacin	1462.27 ± 33.34 #	0	229.00 ± 47.98 *	63.76 ± 7.59	39.83 ± 4.92 *	27.96 ± 8.89
Mature hydrophilic 500	1347.69 ± 46.84 #	6.61 ± 3.25	206.50 ± 68.42 *	67.32 ± 10.83	34.35 ± 1.57 *	37.89 ± 2.84
Mature hydrophilic 1000	1401.76 ± 31.97 #	2.87 ± 2.22	514.67 ± 124.17 #	18.55 ± 19.65	51.24 ± 3.40 #	7.34 ± 6.15

Each column represents the mean + SEM (*n* = 6). Symbol (*) indicates a significant difference (*p* < 0.05) compared to the control group, while symbol (#) indicates that there is no significant difference (*p* > 0.05) compared to the control group, determined by Tukey’s post hoc test.

**Table 5:** Oxidative stress in the exudate.

Group (n).	MDA		GSH		NO		CAT	
	nmol/mL	Inhibition (%)	nmol/mL	Inhibition (%)	µg/mL	Increase (%)	U/mL	Increase (%)
Normal	15.94 ± 0.73 *	-	8.13 ± 0.09 *	-	0.12 ± 0.01 *	-	3.38 ± 0.57 *	-
Control	2138.65 ± 6.64	-	8.74 ± 0.10	-	0.01 ± 0.00	-	6.19 ± 0.82	-
Indomethacin	1963.47 ± 23.34 #	8.19 ± 1.09	8.69 ± 0.12 #	0.64 ± 0.12	0.30 ± 0.02 *	2419.98 ± 171.98	8.64 ± 0.29 *	39.68 ± 4.61
Mature hydrophilic 500	1920.73 ± 104.00 #	10.19 ± 4.86	7.88 ± 0.16 *	7.88 ± 0.16	0.11 ± 0.01 *	834.20 ± 104.04	7.31 ± 0.51 #	18.18 ± 8.21
Mature hydrophilic 1000	2139.14 ± 91.78 #	0	7.71 ± 0.06 *	11.81 ± 0.06	0.13 ± 0.02 *	959.72 ± 146.87	6.19 ± 0.31 #	0

Each column represents the mean + SEM (*n* = 6). Symbol (*) indicates a significant difference (*p* < 0.05) compared to the control group, while symbol (#) indicates that there is no significant difference (*p* > 0.05) compared to the control group, determined by Tukey’s post hoc test.

**Table 6:** Histopathological description of the skin in the scapular region of rats.

	100×	400×	Description
**Normal**	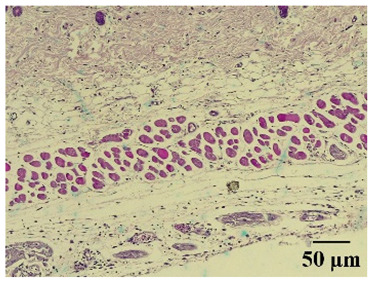	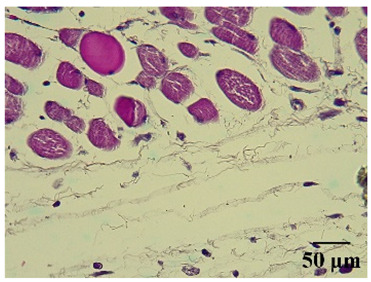	Minimal leukocyte infiltration in the dermis, hypodermis, and subcutaneous tissue. Minimal leukocyte infiltration and slight alteration in the air pouch membrane thickness.
**Control**	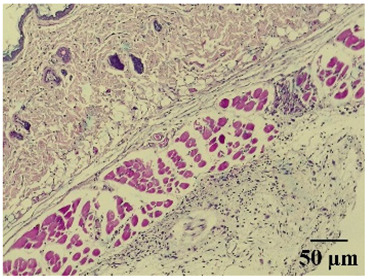	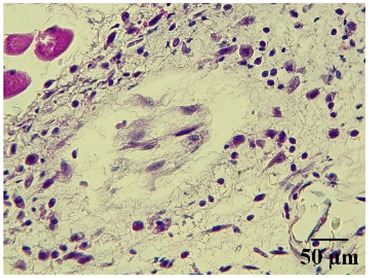	Moderate presence of erythrocytes in subcutaneous tissue. Moderate leukocyte infiltration in the dermis, hypodermis, and muscle layer. Presence of eosinophils at the subcutaneous and muscular levels. Abundant leukocyte infiltration and intense alteration of the air pouch membrane thickness.
**Indomethacin**	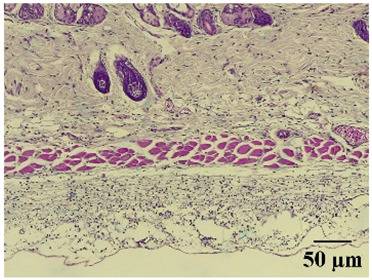	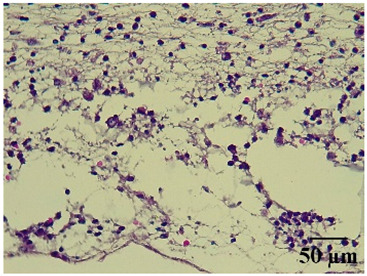	Moderate leukocyte infiltration in the dermis and subcutaneous tissue. Moderate leukocyte infiltration and moderate alteration in the thickness of the air pouch membrane.
**Mature hydrophilic 500**	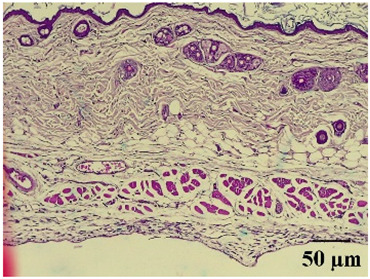	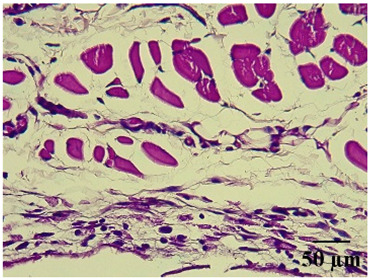	Low leukocyte infiltration in the dermis and subcutaneous tissue. Low leukocyte infiltration and slight alteration in the thickness of the air pouch membrane.
**Mature hydrophilic 1000**	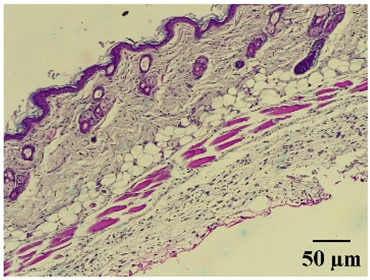	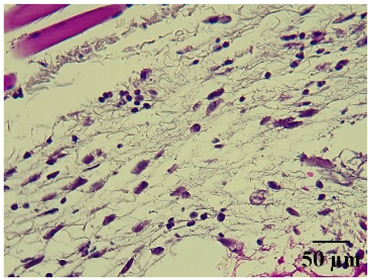	Moderate infiltration of leukocytes in the dermis and subcutaneous tissue. Moderate leukocyte infiltration and moderate alteration in the thickness of the air pouch membrane.

The sections were stained with H&E. The visualization of each image was performed at 100× and 400× magnifications.

## Data Availability

This publication contains all available data.

## Supplementary Materials

The following supporting information can be downloaded at https://www.mdpi.com/article/10.3390/molecules29204964/s1: Figure S1: Percentage of radical reduction in DPPH and ABTS•⁺ at different concentrations of each type of extract; Figure S2: Effect of hydrophilic and lipophilic extracts on the size of the edema; Figure S3: Exudate Volume; Figure S4: Inflammatory cytokines in the exudate; Figure S5: Oxidative stress in the exudate.
