# Supplementary material for: Evaluation of Bioactive Compounds, Antioxidant Capacity, and Anti-Inflammatory Effects of Lipophilic and Hydrophilic Extracts of the Pericarp of Passiflora tripartita var. mollissima at Two Stages of Ripening
Source: Molecules. 2024 Oct 21;29(20):4964. doi: 10.3390/molecules29204964 (PMC11510094; doi:10.3390/molecules29204964)
Supplement: Supplementary file 1 [file molecules-29-04964-s001.zip › molecules-3251832-supplementary.pdf]

molecules

MDPI

Supplementary data

Phytochemical Analysis

Figure S1 shows that as the concentration of extract increases, the percentage of DPPH and **ABTS•+** radical reduction also increases.

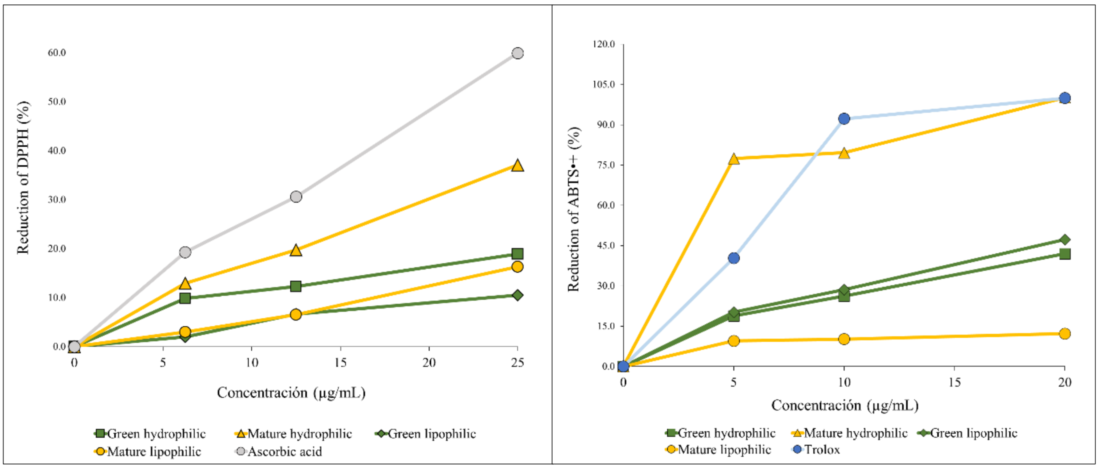

A B

Figure S1. Percentage of radical reduction in DPPH and ABTS•+ at different concentrations of each type of extract.

Anti-inflammatory effect on plantar edema

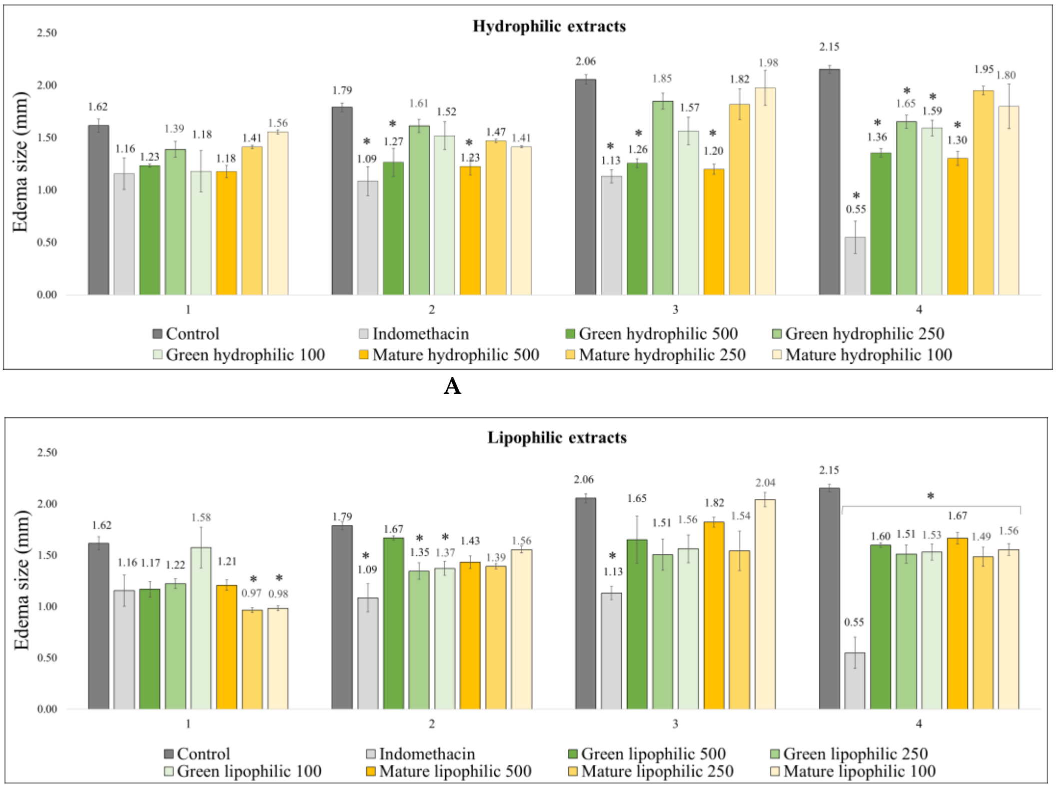

A

B

Figure S2. Effect of hydrophilic and lipophilic extracts on the size of the edema.

Data are presented as mean ± SEM (n = 6). The symbol (*) indicates a significant difference ($p < 0.05$) compared to the control group determined by Tukey's post hoc test.

Anti-inflammatory effect on carrageenan-induced air pouch

Exudate Volume

Figure S3 shows that mature hydrophilic extract at 500 mg/kg significantly reduced exudate volume ($p < 0.05$) compared to the control group and showed a similar effect to the blank group ($p = 0.567$).

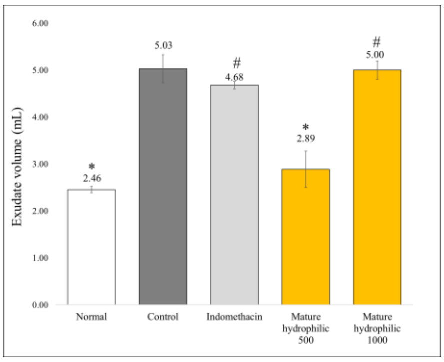

Figure S3. Exudate Volume

Data are presented as mean $\pm$ SEM (n = 6). Symbol (*) indicates a significant difference ($p < 0.05$) compared to the control group, while symbol (#) indicates that there are not significant difference ($p > 0.05$) compared to the control group, determined by Tukey's post hoc test

Inflammatory cytokines in exudate

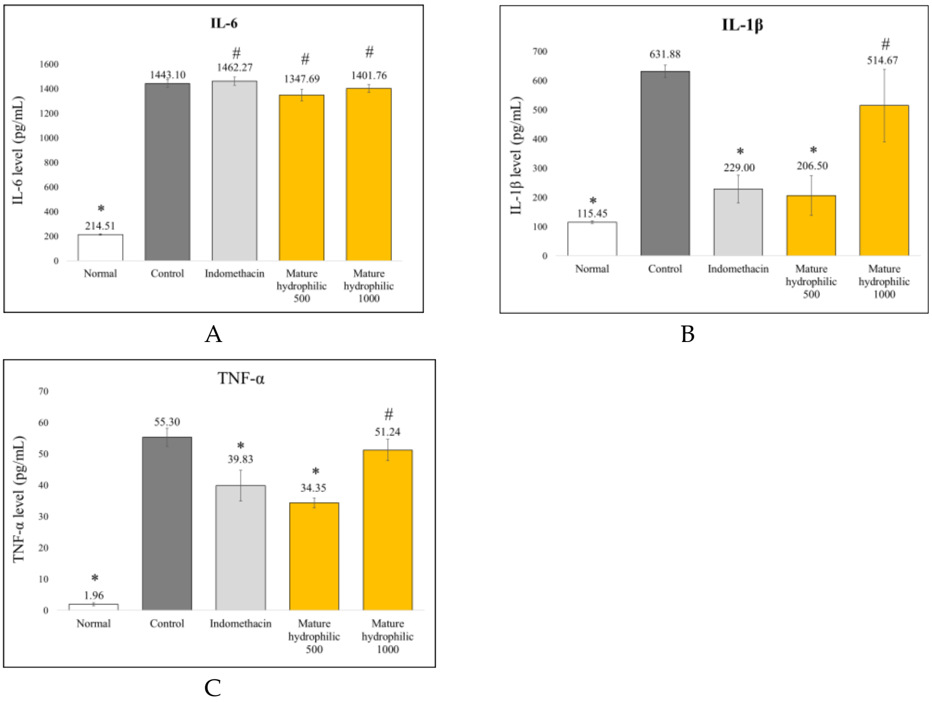

Figure S4. Inflammatory cytokines in exudate.

IL-6: Interleukin 6; IL-1β: Interleukin 1 beta; TNF-α: Tumor necrosis factor alpha.
Data are presented as mean $\pm$ SEM (n = 6). Symbol (*) indicates a significant difference ($p < 0.05$) compared to the control group, while symbol (#) indicates that there are not significant difference ($p > 0.05$) compared to the control group, determined by Tukey's post hoc test.

Oxidative stress in exudate

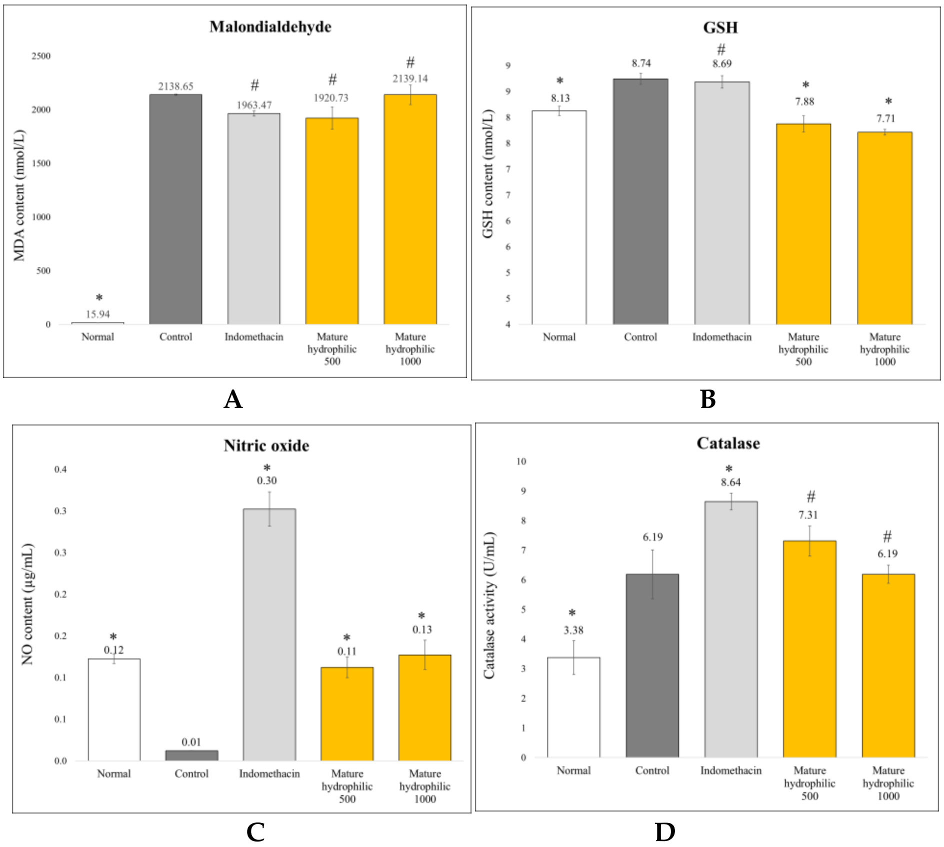

Figure S5. Oxidative stress in exudate.

Data are presented as mean ± SEM (n = 6). Symbol (*) indicates a significant difference ($p < 0.05$) compared to the control group, while symbol (#) indicates that there are not significant difference ($p > 0.05$) compared to the control group, determined by Tukey's post hoc test.
